# Adaptive Resolution Enhancement for Visual Attention Regions Based on Spatial Interpolation

**DOI:** 10.3390/s23146354

**Published:** 2023-07-13

**Authors:** Zhixuan Zhu, Xin He, Chunlai Li, Shijie Liu, Kun Jiang, Kang Li, Jianyu Wang

**Affiliations:** 1 Hangzhou Institute for Advanced Study, University of Chinese Academy of Sciences, Hangzhou 310024, China; zhuzhixuan21@mails.ucas.ac.cn (Z.Z.); lichunlai@mail.sitp.ac.cn (C.L.); liushijie@ucas.ac.cn (S.L.); jiangkun211@mails.ucas.ac.cn (K.J.); likang222@mails.ucas.ac.cn (K.L.); 2 Key Laboratory of Space Active Opto-Electronics Technology, Shanghai Institute of Technical Physics, Chinese Academy of Sciences, Shanghai 200083, China; 3 University of Chinese Academy of Sciences, Beijing 100049, China

**Keywords:** super resolution, eye tracking, visual attention region, virtual reality

## Abstract

Resolution enhancement is crucial for human vision. However, it can be resource-consuming in the display pipeline. Therefore, there is a need to develop a lightweight resolution improvement algorithm specifically targeting visual attention regions. This paper presents a spatial-interpolation-based algorithm to improve the resolution of the visual attention area. The eye-tracking system consists of a near-infrared camera and an event camera is proposed to obtain the 3D gaze vector and eye moving trajectory. Secondly, the observation coordinates are obtained by gaze vectors, and the visual attention region is defined by the sensitive field-of-view angle. Then, interpolation-based adaptive spatial resolution enhancement and contrast enhancement adjustment are performed in the visual attention area. Finally, the feasibility of the proposed method is tested on both qualitative and quantitative dimensions. The experimental results demonstrate that the proposed method can significantly improve the visual effects.

## 1. Introduction

Super-resolution is an image processing technique that transforms low-resolution images into high-resolution ones through certain methods [1,2,3,4]. Interpolation algorithm is a traditional super-resolution reconstruction method [5]. It resamples the low-resolution image, extracts useful information to reconstruct the image, and achieves the purpose of enlarging the image and obtaining a higher resolution. It can effectively improve the quality and details of images. In augmented reality (AR) and virtual reality (VR) applications, high resolution is particularly important for improving the user’s visual experience [6,7,8,9]. However, many existing AR/VR devices are limited by hardware conditions, and thus the image quality and resolution cannot satisfy the visual sensory needs of users. Therefore, there is an urgent need to develop lightweight, hardwareized super-resolution technology to solve this problem.

Some recent studies on super-resolution reconstruction are based on deep learning methods. VDSR [10], LapSRN [11], SR-LUT [12] and other deep-learning algorithms have achieved good results. However, the computational cost and GPU memory usage of deep-learning-based super-resolution algorithms are very high, which are difficult to deploy in mobile devices, such as AR/VR through hardware.

In the AR/VR system, visual-attention-area-oriented super-resolution takes the characteristics of the sensitive field of view into consideration [13]. While using the AR/VR devices, the user’s sight is usually focused on a certain area in the image, which is the so-called “visual attention regions”. Performing the super-resolution algorithm on the visual attention region [14,15,16,17] could meet customers’ vision expectations. To achieve this goal, the user’s gaze and the current fixation point location could be determined by an eye-tracking system. The commonly used sensors to obtain annotation information are infrared cameras and event cameras. The system in this paper adopts multi-sensor fusion. The infrared camera and the event camera are used to obtain the annotation information and eye movement information, and they constitute the eye-tracking system, which feeds back the captured annotation information to the display to determine the staring area. Then, the spatial-based interpolation and contrast enhancement algorithms are applied on the visual attention region. Performing super-resolution on local areas can reduce computation and save resources while achieving better visual effects.

The main contributions of this article are as follows:

(1) An adaptive spatial resolution improvement algorithm is proposed, which uses the traditional interpolation algorithm. The algorithm achieves better performance and reduces the computation.
(2) Contrast enhancement and sharpening are carried out to further improve the visual effect of the image after the over-division.
(3) The super-resolution algorithm is combined with eye movement information, and the resolution of the visual attention area is improved, which improves the visual experience of human–computer interaction.
(4) This algorithm adopts a non-deep-learning method and could be implemented in hardware due to its low computational cost.

The rest of this article is organized as follows. The second part introduces the background and motivations of the research. The third part presents the process of the super-resolution algorithm, contrast enhancement algorithm, eye movement and annotation information acquisition in detail. The fourth part lists the experimental results and verifies the effectiveness of the algorithm. Lastly, the fifth part gives the conclusion of this study and the future work.

## 2. Related Work and Motivations

AR and VR technologies are among the most widely discussed and researched areas in the field of technology. Their applications cover many fields, including gaming, healthcare, manufacturing, entertainment and so on. However, one challenge that AR/VR systems face is the need for high-resolution displays that can provide a realistic and detailed experience. The image processing and display technology in AR/VR are crucial, as they directly impact the user’s perception of the virtual scene.

Super-resolution reconstructions are mainly divided into three types: reconstruction-based methods, interpolation-based methods, and learning-based methods. Interpolation algorithms can be divided into two categories: spatial-based interpolation and frequency-based interpolation. Traditional spatial-based interpolation algorithms include bilinear interpolation [18], bicubic interpolation [19], and nearest-neighbor interpolation [20]. Common frequency-based interpolation algorithms include wavelet-based interpolation [21] and locally linear-embedding-based interpolation [22]. Interpolation [23] is a commonly used super-resolution algorithm that can effectively improve the quality and details of images. The advantages of interpolation algorithms are their simple implementation and low computational complexity. However, they may introduce noise, blur image details, and change the color and contrast of images, resulting in image distortion [24]. To address these issues, researchers have proposed improved interpolation algorithms, such as the improved algorithm of bilinear interpolation [25], which can effectively reduce noise and improve image quality and details. A method based on reconstruction requires prior information to constrain the reconstruction process. When processing image tasks with large amplification coefficient, the performance of the algorithm will become poor due to the lack of prior information. Dong et al. [26] proposed the first model to reconstruct HR images using a convolutional neural network (CNN) approach, with a result that was better than some traditional methods. Simonyan K et al. [10] proposed Very Deep Convolutional Networks for super-resolution; although the result was satisfactory, its network layers were too deep. Jo Y et al. [12] proposed super-resolution using a look-up table; this was a fast super-resolution method, but the effect was not obvious.

The visual-attention-area oriented super-resolution algorithm can reduce computation and alleviate the computational complexity. An eye-tracking system is needed to realize the visual-attention-area-oriented super-resolution. The eye-tracking system includes an event camera [27,28] and an infrared camera. Event cameras are used to obtain eye movement information and infrared cameras are used to obtain fixation points. Since only the regions of interest to the user need to be super-resolution-processed, this saves computational resources. Visual-attention-area-oriented super-resolution rendering technology can be widely used in AR/VR systems in the future.

## 3. System and Methods

**Figure 1:**
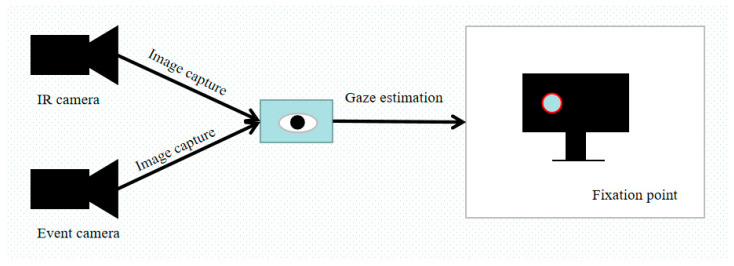
The structural diagram of the experimental device.

The whole system consists of an event camera, an infrared camera and a display device. The whole eye-tracking system is placed in front of a display device and should be lower than the height of the display device to avoid interference caused by occlusion of the display device. The structural diagram of the experimental system is shown in Figure 1.

The infrared camera is a device used to obtain eye-tracking data and fixation points. It has the advantages of low cost, small size and being easy to use. Its principle is to detect the position and movement of the eye through the reflection of infrared light, and then infer the fixation point of the eye. Specifically, the infrared camera emits infrared light, which is reflected by the eye, and the camera captures the reflected light. Then, image-processing algorithms are used to analyze the position and movement of the reflected light. By comparing the eye positions captured at different times, the camera can calculate the trajectory and fixation point of the eye.

**Figure 2:**
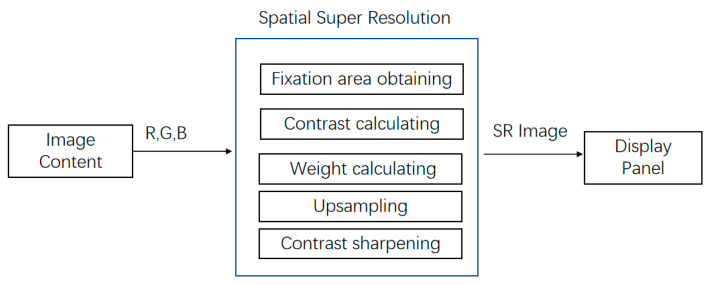
The flowchart of the super-resolution algorithm.

As mentioned above, the method proposed in this article is mainly used to improve the resolution of the fixation area, achieving eye-tracking interaction [29] with super-resolution technology. The framework of our algorithm includes three parts: gaze acquisition, super-resolution reconstruction and contrast enhancement. Super-resolution reconstruction includes three steps: calculating contrast, calculating weight and upsampling. The total process can be divided into five steps as in Figure 2.

The steps of Figure 2 are as follows: Firstly, the visual attention area is obtained by the sensor, the contrast of the area is calculated, the weights in the upsampling process of Lanczos interpolation are calculated, the upsampling is carried out and the contrast sharpening is finally improved.

### 3.1. Gaze Vector Acquisition

The gaze vector refers to the vector from the eye to the viewing point, which is used to determine the user’s gaze direction and fixation position. Firstly, in the initial calibration phase, the user fixates on the area identified in advance (such as the red circle in Figure 1) on the screen; the infrared camera and the event camera are used to capture the gaze vector at this time. The transformation matrix between the gaze vector obtained by the eye tracker device in the user coordinate system and the gaze vector of the display plane is obtained. Secondly, the eye-tracking device will perform the corresponding matrix transformation for each gaze vector to obtain the unique display gaze vector. The gaze vector is calculated using the following formula:

(1) $${\vec{v}}_{gaze}={\vec{p}}_{fixation}-{\vec{p}}_{eye}$$

where ${\vec{v}}_{gaze}$ represents the gaze vector, ${\vec{p}}_{fixation}$ represents the coordinates of the fixation point and ${\vec{p}}_{eye}$.represents the coordinates of the eye. The gaze vector is normalized, and the direction of this vector is the gaze direction. After the gaze vector is projected, the two-dimensional coordinate value in the display coordinate system is obtained, which is the gaze coordinate, represented as the point the user is looking at during the eye interaction.

### 3.2. Adaptive Spatial Resolution Enhancement Module

The super-resolution reconstruction algorithm used in this paper uses a 4 × 4 filter with 12 core pixels and presents an oval shape. The final filtering kernel is generated iteratively using bilinear interpolation. The core idea was to use the Lancozs-like function for upsampling. The Lanczos function is essentially a sinc function that can be used in resampling algorithms, and theoretically, it is an optimal reconstruction filter window function. Moreover, its length and window are adaptive, and it has good anti-aliasing characteristics. Therefore, we introduced the locally adaptive elliptical Lanczos-like filter into our spatial resolution enhancement algorithm and applied it separately in the horizontal and vertical directions [30].

**Figure 3:**
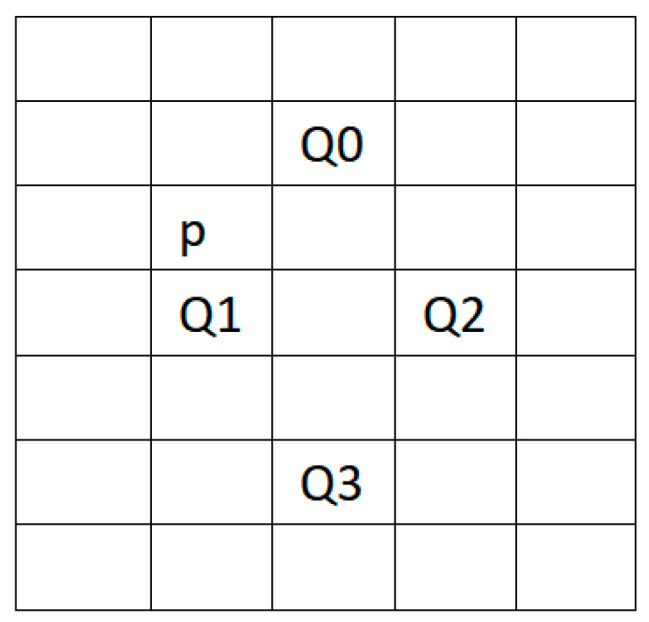
The coordinate relationship of each pixel.

We facilitated every point on the matrix of target resolution sizes, calculated the coordinate mapped to the low-resolution (LR) image and obtained the pixels and texture information near the coordinate, which were used to calculate texture information and resampling. The calculated LR image coordinate pixel point, obtained by rounding down the coordinate value P, was stored as the integer and decimal parts. The coordinates of p + (1, −1), p + (0, 1), p + (2, 1) and p + (1, 3) are, respectively, denoted as Q0, Q1, Q2 and Q3. The coordinate relationship of each pixel is shown in Figure 3.

Edge-detection algorithms [31,32] typically calculate the gradient magnitude and direction of each pixel, and determine whether it is an edge point by comparing the gradient magnitude with a threshold. The two-dimensional direction gradient vector, *dir*, represents the gradient value in the corresponding direction [33]. The horizontal and vertical gradient values of each pixel are calculated, and the two-dimensional gradient vector is obtained. The magnitude and direction of the vector could be used to represent the edge features of the pixel. The length value, *len*, of the two-dimensional direction gradient vector represents the magnitude or strength of the gradient vector. The gradient magnitude represents the local variation or gray level slope of each pixel in the image, and a larger magnitude indicates a more drastic change and is usually associated with edge or corner features. If the gradient magnitude exceeds a predefined threshold, it is considered as an edge point; otherwise, it is considered as a non-edge point. The two-dimensional gradient vector, *dir*, *len*, can be initialized. The formula for calculating the brightness value of each pixel is:

(2) luminance=0.5∗R+0.5∗B+G

where *R*, *G* and *B* are the values of different channels of pixel, respectively. Edges are typically composed of pixels with large brightness variations. Taking the coordinate of Qi and pixel brightness as inputs, the weight, w, is accumulated iteratively using bilinear interpolation. The parameters required for the class Lancozs interpolation function are calculated based on the position of the four points. The horizontal and vertical gradient magnitudes and vectors are calculated separately, denoted as *lx*, *dx* and *ly*, *dy*, respectively. *lx* and *ly* are multiplied by the weight value, w, and added to the gradient magnitude, *len*, while the gradient values in each direction are multiplied by w and added to the horizontal and vertical direction gradient vectors. The gradient values and length values of *dir* are iteratively calculated for each pixel, and the above results are weighted and summed according to w to obtain the final gradient vector and length.

Different upsampling methods are employed for edge and non-edge regions. For non-edge regions, the weight values of each point are computed and processed using weighted averaging. For edge regions, weighted averaging leads to motion blur, at which point high-pass filtering is required.

Each sampling point corresponding to the input image is interpolated using a Lanczos-like function. The continuous analog signal Lanczos4 function is expressed as:

(3) $$L\left(x\right)=\frac{4\sin\left(\pi x\right)\sin\left(\frac{\pi x}{4}\right)}{\pi^{2}x^{2}}$$

The continuous analog signal of the Lanczos-like function is discretized and fitted by a fourth-order polynomial. The shape of the function is controlled by the range of the control variable ω. The fitting function is:

(4) $$\;\;\;\;L\left(X\right)=\left[\frac{25}{32}x^{4}-\frac{25}{16}x^{2}+\frac{1}{16}\right]{\left(\omega x^{2}-1\right)}^{2}$$

The coefficient of the fourth-order term in the polynomial is used to control the edge features of the sampling function. The edge features are calculated by taking the pixel *Q* and its neighboring pixels in the horizontal and vertical directions, and the resulting feature value is denoted as:

(5) $$E=\left({EX}^{2}+{EY}^{2}\right)$$

(6) $$\;EX=\frac{\left|g\left(Q_{x-1,y}\right)-g\left(Q_{x+1,y}\right)\right|}{\max\left(\left|g\left(Q_{x-1,y}\right)-g\left(Q_{x,y}\right)\right|,\left|g\left(Q_{x+1,y}\right)-g\left(Q_{x,y}\right)\right|\right)}$$

(7) $$\;\;\;EY=\frac{\left|g\left(Q_{X,y-1}\right)-g\left(Q_{x,y+1}\right)\right|}{\max\left(\left|g\left(Q_{x,y-1}\right)-g\left(Q_{X,y}\right)\right|,\left|g\left(Q_{x,y+1}\right)-g\left(Q_{X,y}\right)\right|\right)}$$

The edge feature *E* is calculated for each pixel *Q* and its neighboring pixels in the horizontal and vertical directions. The feature is then normalized to [0, 1] using:

(8) $$Edge={\left(\frac{E}{2}\right)}^{2}$$

The linear relationship between the weight ω and the edge feature *Edge* is established as:

(9) $$\omega =\frac{1}{2}-\frac{1}{4}Edge$$

The edge feature *E* and the weight *ω* are calculated for each of the four cross-shaped regions in the convolution kernel, and the *ω* is bilinearly interpolated with respect to the coordinate position. The resulting *ω* is then used for spatial upsampling at the corresponding sampling point. The horizontal and vertical gradients are calculated and normalized to a direction vector as:

(10) $$dir=\left(cos\alpha ,sin\alpha \right)$$

The direction of the fastest grayscale value change is determined, and the gradient at the sampling point is rotated accordingly. The anisotropic length after rotation is computed, and the coordinate transformation (x·cosα + y·sinα, −x·sinα + y·cosα) is applied as to adapt to edges of different angles. The rotated and scaled RGB or RGBA pixel values are calculated and filled into the target matrix template to generate the super-resolved image using the super-resolution algorithm.

### 3.3. Contrast Enhancement

Firstly, a Gaussian filter is applied to the image generated by the super-resolution algorithm to smooth out high-frequency noise and to perform a color space conversion [32]. Then, a 3 × 3 filter is used to calculate the average contrast in the surrounding region of each pixel to determine the contrast level of the area where the pixel is located. For each pixel, the mean and standard deviation of its surrounding 3 × 3 pixels are calculated, and the contrast of the pixel is determined based on the mean and standard deviation using the formula:

(11) $$\;\;contrast=\frac{pixel_{value}-pixel_{mean}}{k\times pixel_{std}+epsilon}$$

Here, $pixel_{value}$ is the pixel value, $pixel_{mean}$ is the mean of surrounding pixels, std is the standard deviation of surrounding pixels, *k* is a parameter that can be adjusted to control the response range of the contrast and epsilon is a constant that goes to zero infinitely to avoid division by zero.

The image is divided into different regions based on local contrast values. For regions with low contrast, the sharpness of the image is enhanced to improve its clarity and detail. The calculation formula is as follows:

(12) $$I^{\prime }\left(x,y\right)=\frac{I\left(x,y\right)-\mu }{1+k\left(\frac{\sigma }{\tau }-1\right)}$$

The formula implements contrast enhancement, where *I*(*x*, *y*) represents the brightness value of the pixel (*x*, *y*) in the original image, *μ* and *σ* represent the mean and standard deviation of the current region and *k* and *τ* are adjustment parameters. The numerator subtracts the mean value of the current region, and the denominator 1 + *k*(*σ*/*τ* − 1) is an adaptive gain that adjusts according to the contrast of different regions.

High-contrast regions maintain the original sharpness of the image, avoiding excessive contrast enhancement that can amplify noise and produce artifacts. The weight of each pixel in the final image is determined by its local contrast values. The original image is blended with the locally contrast-enhanced image using a weighted average, preserving the details and colors of the original while improving clarity and contrast. The sharpness coefficient *α* can be customized to achieve the desired degree of sharpening. Sharpness α is in the range [0, 1]. The output image is given by:

(13) $$\;\;\;Output=\left(1-\alpha \right)\times SI+\alpha \times OI$$

Here, *SI* is the sharpened image and *OI* is the original image. When *α* is 1, the output image is identical to the original; when *α* is 0, the output image is the sharpened image.

### 3.4. Evaluation Index

We used the peak signal-to-noise ratio (PSNR) and structural similarity (SSIM) to evaluate the results. The PSNR [34] is calculated as:

(14) $$\;\;\;\;PSNR=10{log}_{10}\frac{{\left({MAX}_{I}\right)}^{2}}{MSE}$$

(15) $$\;\;\;MSE\left(x,y\right)=\frac{1}{H\times W}\sum_{i=1}^{H}\sum_{J=1}^{W}{\left[X\left(i,j\right)-Y\left(i,j\right)\right]}^{2}$$

where ${MAX}_{I}$ is the maximum possible value of the pixel value in the image, usually 255; *MSE* is the mean square error; *H* and *W* represent the height and width of the given image; and *X*(*i*, *j*) and *Y*(*i*, *j*) represent the sizes of the pixels corresponding to the real high-resolution image and generated super-resolution image. The SSIM [35] value is:

(16) $$SSIM(x,y)=\frac{\left(2\mu_{x}\mu_{y}+C_{1})(2\sigma_{xy}+C_{2}\right)}{\left({\mu_{x}}^{2}+{\mu_{y}}^{2}+C_{1})({\sigma_{x}}^{2}+{\sigma_{y}}^{2}+C_{2}\right)}$$

where *x* and *y* represent the original image and the processed image, $\mu_{x}$ and $\mu_{y}$ represent the mean value of *x* and *y*, respectively, ${\sigma_{x}}^{2}$ and ${\sigma_{y}}^{2}$ represent the variance of *x* and *y*, respectively, $\sigma_{xy}$ represents the variance of *x* and *y* and $C_{1}$ and $C_{2}$ are two constants, used to avoid denominator-free 0 case.

Mean opinion score (MOS) [36] is a measure used in the domain of Quality of Experience and telecommunications engineering, representing overall quality of a stimulus or system. It is the arithmetic mean over all individual “values on a predefined scale that a subject assigns to his opinion of the performance of a system quality”. Such ratings are usually gathered in a subjective quality evaluation test. It was scored by the tester according to their subjective impression of the test sets in this paper.

## 4. Experiment and Analysis

This section includes three parts: the first part describes the design of the experimental system, the second part validates the effectiveness of the super-resolution algorithm and the third part simulates and implements the eye-tracking interactive super-resolution technology.

### 4.1. Evaluation of Super-Resolution Algorithms

In order to verify the validity and reliability of the proposed super-resolution algorithm, quantitative and qualitative verification were carried out, respectively. From a quantitative point of view, our super-resolution algorithm adopts two evaluation indexes: peak signal noise (PSNR) and result similarity (SSIM). The high-resolution original image (HR) in the dataset was downsampled according to the bicubic method, and the horizontal and vertical directions were downsampled according to 0.5 and 0.25 coefficients, respectively, to obtain the corresponding low-resolution image data set (LR). The images were reconstructed according to our upsampling super-resolution algorithm to obtain the corresponding super-resolution (SR) images. The above evaluation indexes were used to evaluate the super-resolution image and the original high-resolution image after the super-resolution algorithm. Traditional super-resolution algorithms, such as bicubic, nearest and bilinear, were used to calculate these two groups of indicators after sampling to the same spatial resolution. At the same time, super-resolution algorithms based on deep learning, such as VDSR [10], were used to improve the resolution of the downsampled images by the same multiple as the control group to calculate the two groups of indicators. The final PSNR and SSIM were obtained by averaging the indexes of each group in the data set. In the process of ×2 super-resolution reconstruction, the PSNR and SSIM of the proposed algorithm and other algorithms are shown in Table 1.

In the process of ×4 super-resolution reconstruction, the PSNR and SSIM of the proposed algorithm and other algorithms are shown in Table 2.

According to the results in Table 1 and Table 2, the proposed algorithm achieved similar performance as Vdsr in the two evaluation indexes, and released more computing burden than the algorithm based on deep learning.

From a qualitative point of view, we used the subjective mean score (MOS) evaluation index. Multiple groups of super-resolution-reconstructed images were mixed together, including those reconstructed by traditional methods and deep learning methods. The super-resolution algorithm presented in this paper produced better visual effects after 60 testers were asked to select the most visually effective images in each group. The MOS index is shown in Table 3. It can be seen that the method proposed in this paper is effective and reliable.

**Figure 4:**
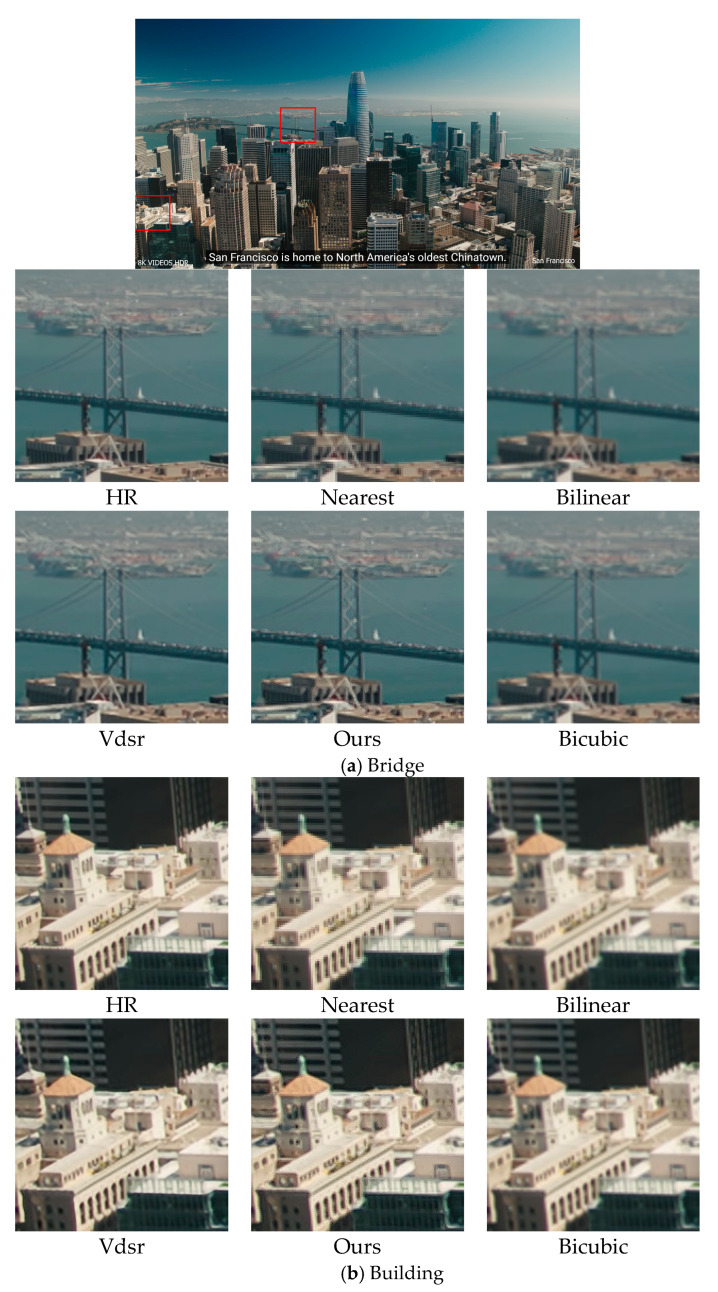

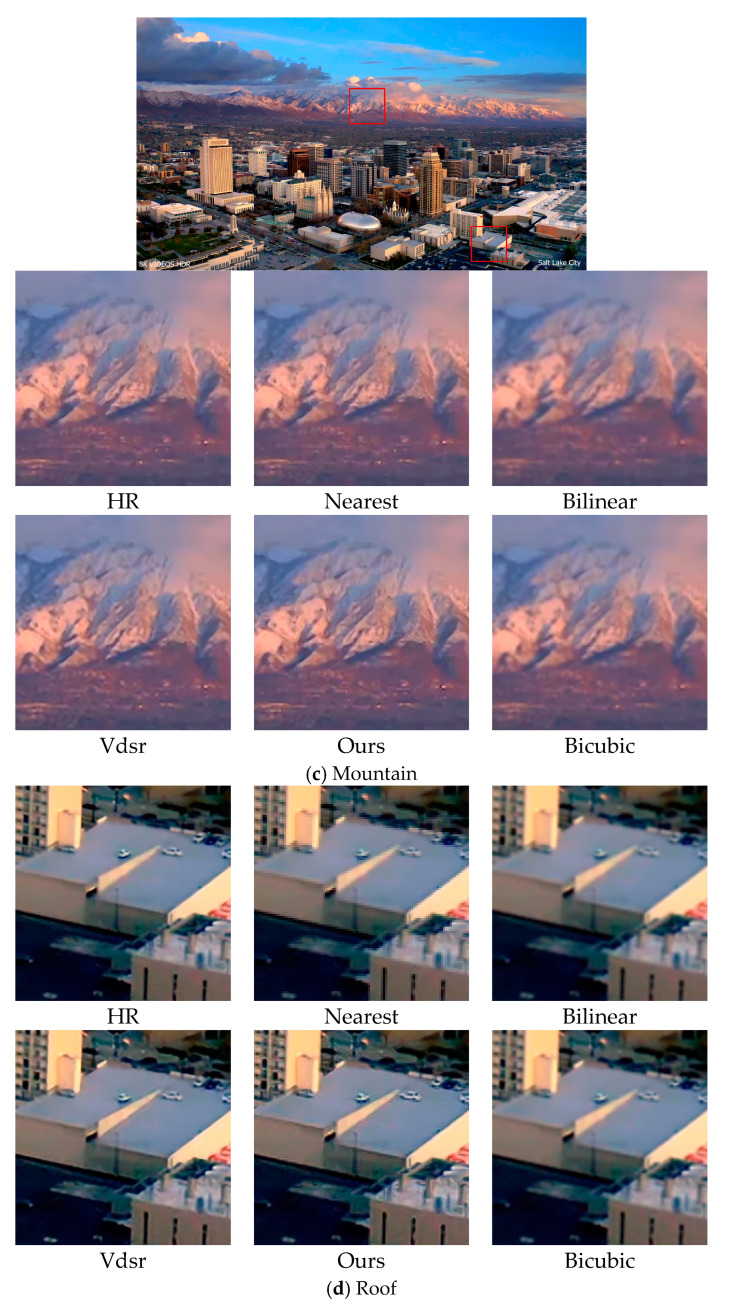

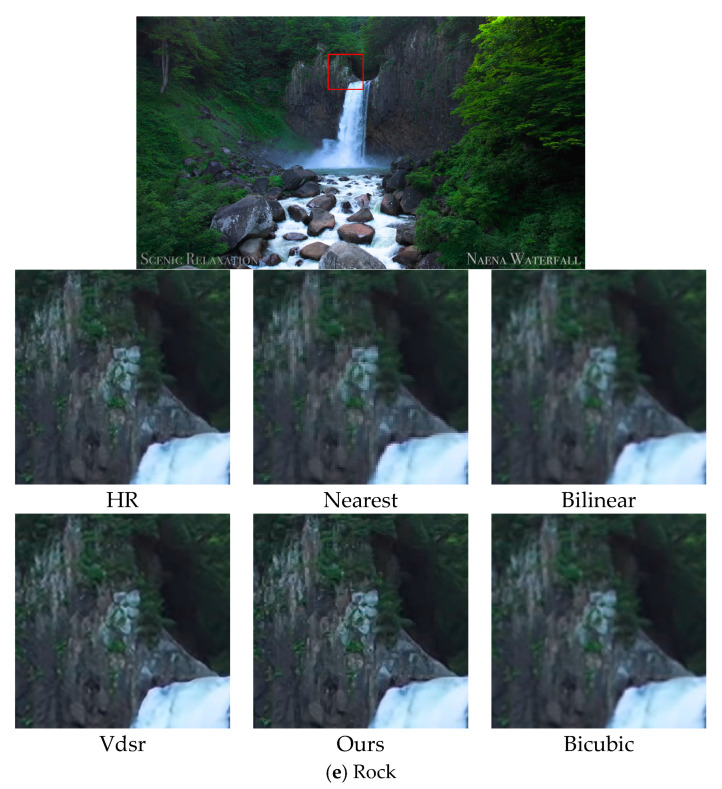
The effect of a single image after the super-resolution algorithm.

The visual difference between our super-resolution reconstruction and other super-resolution reconstructions is shown in Figure 4.

The results showed that our proposed super-resolution algorithm has better visual effects. By using our method, the bridge can be seen more clearly in Figure 4a. The natural scenery in Figure 4c,e is more clearly textured. The details in Figure 4b,d are more reproduced, such as the outline of the car in Figure 4d. Therefore, it can be concluded that the proposed method is more effective and reliable.

### 4.2. Eye-Movement Interaction and Super-Resolution of Fixation Area

#### 4.2.1. Obtain Gaze Area and Eye Movement Trajectory

**Figure 5:**
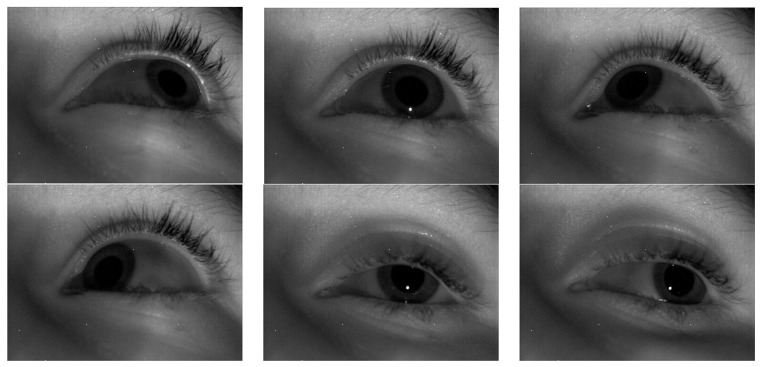
The gaze image of the fixation point captured by the infrared camera.

**Figure 6:**
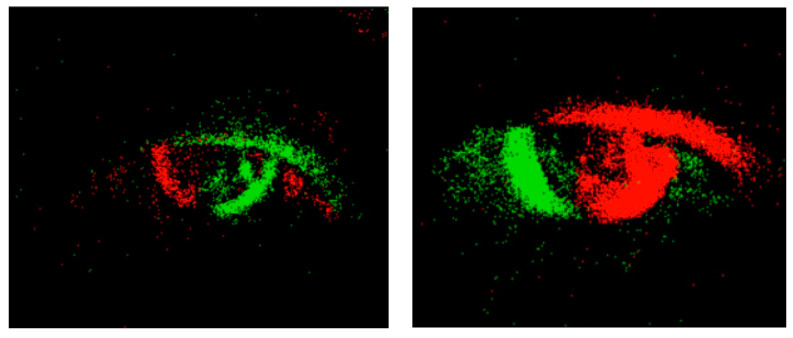
Event camera captures the results of eye motion.

The application scenario of the sensor is to obtain annotation information. The infrared camera captures images of the eye and extracts the position and size of the pupil. Based on the position of the pupil in the image, the direction vector of the eye can be determined. The motion vector of the eye can be obtained by recording the eye’s movement trajectory. Combining the direction vector and motion vector of the eye yields the gaze vector. An event camera is a new type of image sensor that can capture and process visual event information very quickly. Unlike traditional cameras that capture and process images at a fixed frame rate, event cameras individually record changes in light intensity for each pixel in the image sensor and produce event data at a very high time resolution. Event outputs are generated only when the pixel values change. Red and green are usually used to represent different event types or timestamps. Color indicates the change in pixel intensity. This color coding helps to better capture eye-tracking data. The obtained infrared gaze is shown in Figure 5, while the eye-tracking captured by the event camera is shown in Figure 6. Red means pixel brightness increases, green means pixel brightness decreases.

#### 4.2.2. Enhancing the Image Resolution of the Fixation Area

In the near-eye display system, visual-attention-area oriented super-resolution is important. In order to verify the robustness of the algorithm, the elliptical shape annotation trajectory was simulated according to the user’s fixation habit. This simulation upsamples the horizontal and vertical resolution of the central region of the gaze point, achieving a ×4 super-resolution effect, and performs contrast enhancement to highlight edge details. For the outer layer, this simulation only performed contrast enhancement, which can effectively meet the requirements of both super-resolution and visual effects while reducing computational costs and minimizing delays in the eye-movement-tracking process. The human eye has high sensitivity in the 5.2° region of the central retina. Thus, we only need to improve the resolution of the high-sensitivity region. Experiments show that the super-resolution algorithm based on the visual attention area can save about 80% of the computing resource compared with processing the whole image.

**Figure 7:**
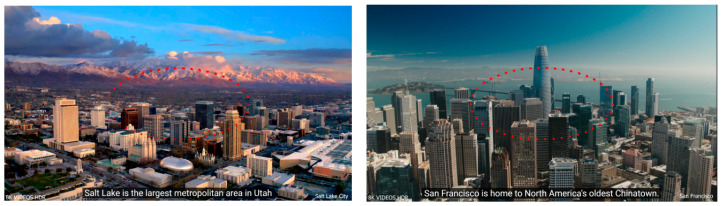
Elliptical gaze trajectory used in simulation.

As illustrated in Figure 7, this simulation employs an elliptical gaze trajectory which is marked in red dots.

According to the simulated elliptic annotation trajectory, the region to be processed is determined.

**Figure 8:**
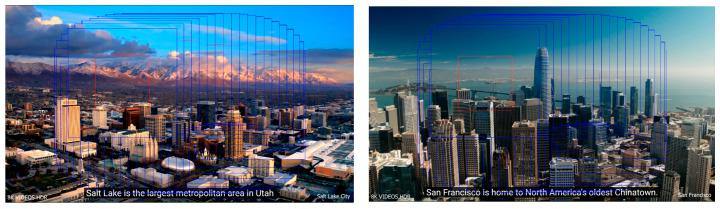
Visual effects after the visual-attention-area oriented super-resolution. The resolution and contrast enhancement were performed in the red rectangle box, and the contrast enhancement was performed in the blue rectangle box outside the red rectangle box.

As long as the eye movement data generated by the user in the process of human–computer interaction is obtained, the trajectory of the gaze point moving on the display can be calculated. The actual gaze trajectory is used to replace the simulated gaze trajectory, and the algorithm can be used in the actual human–computer interaction system. The global simulation results are shown in Figure 8.

**Figure 9:**
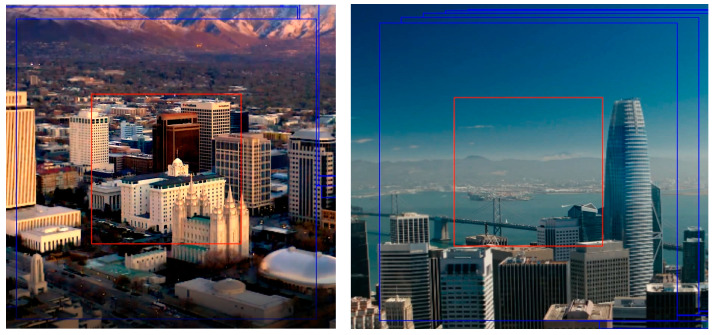
Detailed effects of the visual-attention-area-oriented super-resolution.

The simulation effect under a certain gaze point is shown in Figure 9. The red rectangular box is built around the fixation point that the user is looking at. This part of the region was upsampled to improve the resolution, and at the same time, the contrast adaptive sharpening was performed. Outside the red box, only contrast-adaptive sharpening was performed inside the blue box. Areas other than the blue box represent the original image.

The above figure shows that the proposed eye-tracking-based eye movement interaction super-resolution algorithm is effective and reliable. The edge information of the complex building in the red rectangle is well preserved. The contrast outside the red rectangle box and inside the blue rectangle box is significantly improved compared with outside the blue rectangle box.

The running time of the interpolation algorithm in this paper was compared with other traditional interpolation algorithms, and the experimental results are shown in Table 4. Compared with other traditional algorithms, the proposed algorithm takes only a little longer time. However, the reconstruction effect is significantly better than other traditional effects. The graphics card we used was NVIDIA GeForce RTX4090; the CPU we used was 13th Gen Intel(R) Core(TM) i9-13900K.

Experiments were conducted to test the satisfaction of users of the global execution algorithm and the annotation area execution algorithm. The experimental results are shown in Table 5.

## 5. Conclusions

Resolution enhancement is important for human vision. In this paper, we developed a lightweight resolution enhancement algorithm for visual attention regions. Firstly, the eye-tracking system was proposed to obtain the 3D gaze vector and eye-moving trajectory. Secondly, the observation coordinates were obtained by gaze vectors, and the visual attention region was defined by the sensitive field-of-view angle. Then, interpolation-based adaptive spatial resolution enhancement and contrast-enhancement adjustment were performed in the visual attention area. Finally, the feasibility of the proposed method was tested on both qualitative and quantitative dimensions. The experimental results demonstrate that the proposed method can significantly improve the visual effects. Experiments show that the super-resolution algorithm based on the visual attention area can save about 80% of the computing resource compared with processing the whole image. While the proposed eye-tracking interactive super-resolution algorithm successfully improves resolution based on the gaze area, there is still room for improvement in real-time performance. In the future, the gaze and eye-tracking information acquired by the eye-tracking system will be used to test the performance of this eye-tracking interactive super-resolution algorithm in real time. The algorithm will be deployed in VR/AR devices to test the algorithm performance.

## Figures and Tables

**Table 1:** PSNR and SSIM index values of different algorithms (×2 SR).

Method	PNSR Average	SSIM Average
Bicubic	33.96	0.9763
Nearest	31.19	0.9570
Bilinear	32.40	0.9655
Vdsr	35.33	0.9799
Ours	35.02	0.9793

**Table 2:** PSNR and SSIM index values of different algorithms (×4 SR).

Method	PNSR Average	SSIM Average
Bicubic	27.98	0.8964
Nearest	26.48	0.8635
Bilinear	27.25	0.8825
Vdsr	29.15	0.9160
Ours	28.87	0.9034

**Table 3:** MOS for different test cases.

MOS	Our Method	Other Methods
Building	39	21
Sea	36	24
Code	34	26
Game	41	19
Mountain	40	20
Road	38	22

**Table 4:** Running time for different test cases.

Method	Running Time
Bicubic	42.98 μs
Nearest	23.75 μs
Bilinear	31.74 μs
Ours	61.44 μs

**Table 5:** MOS for different processing area.

**MOS**	**Global**	**Visual Attention Area**
	18	42

## Data Availability

Not applicable.

## References

[1] Tian J., Ma K.K. (2011). A survey on super-resolution imaging. Signal Image Video Process..

[2] Park S.C., Park M.K., Kang M.G. (2003). Super-resolution image reconstruction: A technical overview. IEEE Signal Process. Mag..

[3] Yue L., Shen H., Li J., Yuan Q., Zhang H., Zhang L. (2016). Image super-resolution: The techniques, applications, and future. Signal Process..

[4] Xiao A., Wang Z., Wang L., Ren Y. (2018). Super-resolution for “Jilin-1” satellite video imagery via a convolutional network. Sensors.

[5] Siu W.C., Hung K.W. Review of image interpolation and super-resolution. Proceedings of the 2012 Asia Pacific Signal and Information Processing Association Annual Summit and Conference.

[6] Zhan T., Yin K., Xiong J., He Z., Wu S.-T. (2020). Augmented reality and virtual reality displays: Perspectives and challenges. Iscience.

[7] Xiong J., Hsiang E.L., He Z., Zhan T., Wu S.-T. (2021). Augmented reality and virtual reality displays: Emerging technologies and future perspectives. Light Sci. Appl..

[8] Jensen L., Konradsen F. (2018). A review of the use of virtual reality head-mounted displays in education and training. Educ. Inf. Technol..

[9] Mamone V., Ferrari V., D’Amato R., Condino S., Cattari N., Cutolo F. (2023). Head-Mounted Projector for Manual Precision Tasks: Performance Assessment. Sensors.

[10] Simonyan K., Zisserman A. (2014). Very deep convolutional networks for large-scale image recognition. arXiv.

[11] Lai W.-S., Huang J.-B., Ahuja N., Yang M.-H. Deep laplacian pyramid networks for fast and accurate super-resolution. Proceedings of the IEEE Conference on Computer Vision and Pattern Recognition.

[12] Jo Y., Kim S.J. Practical single-image super-resolution using look-up table. Proceedings of the IEEE/CVF Conference on Computer Vision and Pattern Recognition (CVPR).

[13] Banks M.S., Read JC A., Allison R.S., Watt S.J. (2012). Stereoscopy and the human visual system. SMPTE Motion Imaging J..

[14] Fu W., Wang J., Lu Y., Wu H., Chai X. (2013). Image processing strategies based on visual attention models under simulated prosthetic vision. Zhongguo Yi Liao Qi Xie Za Zhi Chin. J. Med. Instrum..

[15] Baek J., Dosher B.A., Lu Z.L. (2021). Visual attention in spatial cueing and visual search. J. Vis..

[16] Hua Z., Li Y., Li J. Image segmentation algorithm based on improved visual attention model and region growing. Proceedings of the 2010 6th International Conference on Wireless Communications Networking and Mobile Computing (WiCOM).

[17] Kim H., Yang J., Choi M., Lee J., Yoon S., Kim Y., Park W. Eye tracking based foveated rendering for 360 VR tiled video. Proceedings of the 9th ACM Multimedia Systems Conference.

[18] Gribbon K.T., Bailey D.G. A novel approach to real-time bilinear interpolation. Proceedings of the Proceedings. DELTA 2004. Second IEEE International Workshop on Electronic Design, Test and Applications.

[19] Dengwen Z. An edge-directed bicubic interpolation algorithm. Proceedings of the 2010 3rd International Congress on Image and Signal Processing.

[20] Jiang N., Wang L. (2015). Quantum image scaling using nearest neighbor interpolation. Quantum Inf. Process..

[21] Nguyen N., Milanfar P. (2000). A wavelet-based interpolation-restoration method for superresolution (wavelet superresolution). Circuits Syst. Signal Process..

[22] Yang X., Chen Y., Yue X., Ma C., Yang P. (2022). Local linear embedding based interpolation neural network in pancreatic tumor segmentation. Appl. Intell..

[23] Zhang L., Wu X. (2006). An edge-guided image interpolation algorithm via directional filtering and data fusion. IEEE Trans. Image Process..

[24] Liu D., Li Y., Lin J., Li H., Wu F. (2020). Deep learning-based video coding: A review and a case study. ACM Comput. Surv. CSUR.

[25] Sa Y. Improved bilinear interpolation method for image fast processing. Proceedings of the 2014 7th International Conference on Intelligent Computation Technology and Automation.

[26] Dong C., Loy C.C., He K., Tang X. (2015). Image super-resolution using deep convolutional networks. IEEE Trans. Pattern Anal. Mach. Intell..

[27] Scheerlinck C., Rebecq H., Gehrig D., Barnes N., Mahony R., Scaramuzza D. Fast image reconstruction with an event camera. Proceedings of the IEEE/CVF Winter Conference on Applications of Computer Vision (WACV).

[28] Rebecq H., Ranftl R., Koltun V., Scaramuzza D. (2019). High speed and high dynamic range video with an event camera. IEEE Trans. Pattern Anal. Mach. Intell..

[29] Majaranta P., Bulling A. (2014). Eye tracking and eye-based human–computer interaction. Adv. Physiol. Comput..

[30] Madhukar B.N., Narendra R. (2013). Lanczos resampling for the digital processing of remotely sensed images. Proceedings of the International Conference on VLSI, Communication, Advanced Devices, Signals & Systems and Networking (VCASAN-2013).

[31] Kumar M., Saxena R. (2013). Algorithm and technique on various edge detection: A survey. Signal Image Process..

[32] Sun R., Lei T., Chen Q., Wang Z., Du X., Zhao W., Nandi A.K. (2022). Survey of image edge detection. Front. Signal Process..

[33] Nezhadarya E., Ward R K. (2011). A new scheme for robust gradient vector estimation in color images. IEEE Trans. Image Process..

[34] Sara U., Akter M., Uddin M.S. (2019). Image quality assessment through FSIM, SSIM, MSE and PSNR—A comparative study. J. Comput. Commun..

[35] Setiadi D.R.I.M. (2021). PSNR vs SSIM: Imperceptibility quality assessment for image steganography. Multimed. Tools Appl..

[36] Streijl R.C., Winkler S., Hands D.S. (2016). Mean opinion score (MOS) revisited: Methods and applications, limitations and alternatives. Multimed. Syst..

